# Effect of Thyroid Hormones on Kidney Function in Patients after Kidney Transplantation

**DOI:** 10.1038/s41598-020-59178-x

**Published:** 2020-02-07

**Authors:** Benjamin Schairer, Viktoria Jungreithmayr, Mario Schuster, Thomas Reiter, Harald Herkner, Alois Gessl, Gürkan Sengölge, Wolfgang Winnicki

**Affiliations:** 1 Department of Internal Medicine III; Division of Nephrology and Dialysis, Medical University of Vienna, Vienna, Austria; 2 Department of Pharmacology and Toxicology, University of Vienna, Vienna, Austria; 3 Department of Emergency Medicine, Medical University of Vienna, Vienna, Austria; 4 Department of Internal Medicine III, Division of Endocrinology, Medical University of Vienna, Vienna, Austria

**Keywords:** Thyroid diseases, Kidney diseases

## Abstract

Elevated levels of thyroid-stimulating-hormone (TSH) are associated with reduced glomerular filtration rate (GFR) and increased risk of developing chronic kidney disease even in euthyroid patients. Thyroid hormone replacement therapy has been shown to delay progression to end-stage renal disease in sub-clinically hypothyroid patients with renal insufficiency. However, such associations after kidney transplantation were never investigated. In this study the association of thyroid hormones and estimated GFR (eGFR) in euthyroid patients after kidney transplantation was analyzed. In total 398 kidney transplant recipients were assessed retrospectively and association between thyroid and kidney function parameters at and between defined time points, 12 and 24 months after transplantation, was studied. A significant inverse association was shown for TSH changes and eGFR over time between months 12 and 24 post transplantation. For each increase of TSH by 1 µIU/mL, eGFR decreased by 1.34 mL/min [95% CI, −2.51 to −0.16; p = 0.03], corresponding to 2.2% eGFR decline, within 12 months. At selected time points 12 and 24 months post transplantation, however, TSH was not associated with eGFR. In conclusion, an increase in TSH between 12 and 24 months after kidney transplantation leads to a significant decrease in eGFR, which strengthens the concept of a kidney-thyroid-axis.

## Introduction

Interactions between thyroid hormones and kidney function have been suggested in previous studies. Data from animal studies show that thyroid hormones are important players in the development and growth of kidneys^1,2^. This is supported by a study with children suffering from congenital hypothyroidism and renal anomalies^3^. Thyroid hormones also alter glomerular filtration rate (GFR) by affecting renal blood flow and vascular resistance in adult kidneys^4–6^. Large retrospective analyses have shown associations of estimated kidney function with thyroid function, including an inverse association between estimated GFR (eGFR) and prevalence of hypothyroidism^7^, as well as the risk of developing subclinical and overt hypothyroidism^8^. Conversely, hypothyroidism and subclinical hypothyroidism have been found to lead to higher prevalence of chronic kidney disease (CKD) over time^9^. Furthermore, even in the normal reference range low free triiodothyronine (FT3) and resulting high TSH values have been associated with higher risk for the development of CKD^10^. Low thyroid hormone values, even within the clinically normal reference range, have been shown to be associated with reduced eGFR^11^. In addition, TSH quintiles in normal range have been consistently and independently associated with decreased GFR, increased urine albumin/creatinine ratio and CKD^12^. Consequently, it was hypothesized that correction of subclinical hypothyroidism by thyroid hormone replacement therapy (THRT) would stabilize and increase renal function measured by eGFR over time. In a small study with 37 hypothyroid and 14 hyperthyroid patients, den Hollander *et al*. investigated plasma creatinine levels and eGFR before THRT and after regaining euthyroidism; in previously hypothyroid patients renal function improved significantly after THRT. Conversely, kidney function was shown to decrease after treatment for hyperthyroidism^13,14^. Furthermore, thyroid hormone replacement therapy has been shown to preserve renal function and attenuate the decline of eGFR in patients with CKD and subclinical hypothyroidism^15,16^.

Patients with renal grafts by definition have a chronic kidney disease independent of their eGFR. Still, they differ in many aspects from non-transplant patients with CKD. Continuous long-term therapy with nephrotoxic immunosuppressants and corticosteroids interacting with many organ systems, including the thyroid gland, is one of the reasons that makes this population unique. Also, many CKD effects on thyroid function (low-T3 syndrome, subclinical hypothyroidism) normalize after kidney transplantation (KTx)^17^. However, the potential effect of thyroid hormones on GFR or the preservation of renal function in kidney transplanted individuals has not been demonstrated. In this study, we analyzed data from 398 euthyroid CKD patients who received kidney transplants between 2003 and 2016 to investigate the effect of the thyroid function parameters TSH, triiodothyronine (T3), free thyroxine (FT4) and thyroxine (T4) and their dynamics on eGFR course.

## Methods

### Study design and participants

This retrospective cohort study was designed to investigate the effect of thyroid hormone dynamics on renal function parameters. Datasets from 398 kidney transplant patients who received a kidney transplant at the Medical University of Vienna between 2003 and 2016 and were in continuous care at this site were analyzed. Adult patients over 18 years with euthyroid state (TSH 0.44 to 3.77 µIU/mL) 12 months after transplantation were included. Exclusion criteria were age <18 years, pregnancy, malignancy, high dose glucocorticoid treatment with prednisolone >25 mg daily and therapy with lithium, amiodarone or propranolol.

The association between thyroid function, measured by TSH, T3, FT4 and T4 and kidney function, determined by eGFR, was investigated at different time points and over time. First, the association between TSH, T3, FT4 and T4 and eGFR was tested at 12 and 24 months after renal transplantation. In addition, it was examined whether TSH, T3, FT4 and T4 concentration at 12 months after transplantation affects the course of eGFR within the following 12 months (eGFR^24months^ − eGFR^12months^ = ∆eGFR). Subsequently, it was investigated whether a change in TSH, T3, FT4 and T4 levels within 12 months (i.e. TSH^24months^ − TSH^12months^ = ∆TSH) is associated with a change in eGFR over time (∆eGFR). We have conducted several regression models on eGFR at different time points and periods to investigate the association between TSH, T3, FT4 and T4 and eGFR under the aforementioned conditions.

### Laboratory diagnostic

All laboratory routine measurements were carried out at the Department of Laboratory Medicine of the Medical University of Vienna, which is certified (ISO 9001:2008) and accredited (ISO 15189:2008) for quality management. Laboratory testing of TSH, T3, FT4 and T4 was performed by a third generation ultrasensitive electrochemiluminescence immunoassay (Elecsys on Modular and Cobas e 602 instruments, Roche Diagnostics, Penzberg, Germany). Testing of serum creatinine was performed by Jaffe reaction and eGFR was calculated by the chronic kidney disease EPI (CKD-EPI) equation^18^.

### Data processing and statistical analyses

MS Excel (© Microsoft, Redmont, WA) and Stata 14 (Stata Corp, College Station, TX) were used for data management and analyses. Continuous data are presented as mean ± standard deviation; categorized data are presented as absolute count and relative frequencies. Distributional properties of continuous variables were assessed and linear regression models were deployed to estimate the association between thyroid function and renal function parameters. Due to the longitudinal design of the study, we used thyroid function and renal function variables at specific time points or delta values between these points over time to avoid within-individual-correlations.

Multivariable linear regression models were used to adjust the main effect estimates for potential confounding factors. First-level interactions were assessed by stratified analyses and by introducing interaction terms as co-variables into the models. We report regression coefficients for change in unit outcome per one unit change in predictor with 95% confidence intervals. We quantified hypertension per number of antihypertensive substance class prescribed to achieve current ESC/ESH blood pressure targets. A two-sided p-value less than 0.05 was considered statistically significant.

### Approval and registration

The study protocol was performed in accordance to the 1964 Helsinki Declaration and its later amendments and was approved by the Ethics Committee of the Medical University of Vienna (EK 2038/2017). All study participants were adults over 18 years and gave written informed consent. All procedures were performed in accordance with ethical standards and the relevant regulations and guidelines and no organs/tissues from prisoners were procured.

## Results

The demographic and clinical characteristics of the study population are presented in Table 1. Mean age at renal transplantation was 51.6 ± 13.4 years (range 18–77 years). Stratified by gender, 35% study subjects were women (n = 141, mean age 51 years, range 19–72 years) and 65% were men (n = 257, mean age 50 years, range 18–77 years). The mean body mass index was 25.5 ± 4.6 kg/m². At the start of data analysis (12 months after transplantation) all patients were in euthyroid condition and 48 of those were under a thyroid hormone replacement therapy (THRT).

**Table 1:** Baseline characteristics of the study population.

Characteristic	Study subjects (n = 398)
Male sex - number (%)	257 (65)
Female sex - number (%)	141 (35)
Age at renal transplantation - years	51 ± 13
Height - cm	171 ± 9
Weight - kg	75 ± 15
Body Mass Index - kg/m²	26 ± 5
Patients under THRT - number (%)	48 (12)
Patients with arterial hypertension – number (%)	352 (88)
Patients with diabetes mellitus – number (%)	73 (18)

Plus-minus values are means ± standard deviation. Numbers in parentheses indicate percentage. Abbreviations: cm, centimetre; kg, kilogram; THRT: thyroid hormone replacement therapy.

One percent of study subjects had a urine protein/creatinine ratio above 3000 mg/g at 12 months post transplantation, which did not change in the course of the study.

Kidney and thyroid function parameters at baseline (pre KTx), 12 and 24 months after kidney transplantation are presented in Table 2. Twelve months post transplantation, serum creatinine levels ranged from 0.6 to 5.5 mg/dL (mean 1.5 mg/dL) and eGFR from 15 to 104 mL/1.73 m² per minute (mean 52.9 ± 17.0 mL/1.73 m² per minute). Twenty-four months post KTx serum creatinine levels ranged from 0.7 to 4.6 mg/dL (mean 1.5 mg/dL) and eGFR from 11 to 112 mL/1.73 m² per minute (mean 52.9 ± 17.4 mL/1.73 m² per minute). Mean TSH at 12 and 24 months post renal transplantation were 1.74 µIU/mL (range: 0.44 to 3.73 µIU/mL) and 1.82 µIU/mL (range: 0.22 to 7.11 µIU/mL), respectively. Baseline TSH levels were 2.06 ± 0.99 µIU/mL. Corresponding values for T3, FT4 and T4 are shown in Table 2.

**Table 2:** Laboratory tests including kidney and thyroid function parameters 12 and 24 months post kidney transplantation.

Parameter	Pre KTx	12 months post KTx	24 months post KTx
Creatinine (mg/dL)	n.a.	1.5 ± 0.4 [0.6–5.5]	1.5 ± 0.5 [0.7–4.6]
eGFR (mL/1.73 m²/min)	n.a.	52.9 ± 17.0 [15–104]	52.9 ± 17.4 [11–112]
TSH (µIU/mL)	2.06 ± 0.99 [0.06–6.86]	1.74 ± 0.75 [0.44–3.73]	1.82 ± 0.87 [0.22–7.11]
T3 (ng/mL)	0.92 ± 0.25 [0.24–2.26]	0.97 ± 0.20 [0.63–1.47]	0.91 ± 0.20 [0.54–1.38]
FT4 (ng/dL)	1.20 ± 0.25 [0.69–1.93]	1.31 ± 0.19 [0.83–1.64]	1.34 ± 0.25 [0.74–2.09]
T4 (ng/mL)	69.34 ± 16.19 [37–158]	72.69 ± 14.69 [48–121]	71.68 ± 13.59 [53–118]
CRP > 5 mg/dL – number	0 (0%)	4 (1.0%)	3 (0.8%)
Tacrolimus level > 12 ng/mL - number	0 (0%)	6 (1.5%)	5 (1.3%)
BUN/creatinine ratio > 20 - number	n.a.	34 (8.5%)	37 (9.2%)
U: P/C > 3000 mg/g - number	n.a.	1 (0.3%)	3 (0.8%)
Antihypertensive substances used – number*	2.5 ± 1.5	2.2 ± 1.4	2.3 ± 1.4
Patients with diabetes mellitus	73 (18%)	74 (19%)	72 (18%)

Plus-minus values are means ± standard deviation. Numbers in brackets indicate the range, numbers in parentheses indicate percentage.

Abbreviations: BUN, blood urea nitrogen; CRP, C-reactive protein; eGFR, estimated glomerular filtration rate; FT4, free thyroxine; KTx, kidney transplantation; n.a., not applicable; T3, triiodothyronine; T4, thyroxine; TSH, thyroid-stimulating hormone; U: P/C, urine protein/creatinine ratio. *Number of antihypertensive substance class used to achieve current ESC/ESH blood pressure targets.

### Estimated glomerular filtration rate depending on thyroid function

In a linear regression analysis there was no association between single TSH levels and eGFR examined 12 and 24 months after transplantation. Also, single TSH levels were not associated with a change in eGFR between month 12 and month 24 after transplantation (∆eGFR). However, a significant association between ∆TSH and ∆eGFR could be shown between month 12 and 24 post KTx. With each increase of the TSH value by one µIU/mL, the eGFR decreased by 1.41 mL/min [95% CI, −2.56 to −0.26; p = 0.02] corresponding to a 2.2% decline of eGFR within 12 months (Table 3).

**Table 3:** Association between TSH and eGFR at various time points and periods post KTx in all patients (n = 398).

Outcome	Exposure	Regression coefficient (95% CI) unadjusted	p-value	Regression coefficient (95% CI) adjusted*	p-value
eGFR	TSH at 12 mo	0.54 [−1.69 to 2.78]	0.63	0.01 [−2.11 to 2.12]	0.99
	TSH at 24 mo	0.17 [−1.81 to 2.14]	0.87	−0.19 [−2.08 to 1.71]	0.85
∆eGFR	TSH at 12 mo	0.86 [−0.27 to 1.99]	0.13	0.76 [−0.40 to 1.92]	0.20
	∆TSH from 12 to 24 mo	−1.41 [−2.56 to −0.26]	0.02	−1.34 [−2.51 to −0.16]	0.03

∆eGFR = eGFR^24months^ − eGFR^12months^; ∆TSH = TSH^24months^ − TSH^12months^. Numbers in brackets indicate the range.

Abbreviations: eGFR, estimated glomerular filtration rate; KTx, kidney transplantation; TSH, thyroid-stimulating hormone; mo, months. *Adjusted for age, gender, BMI, THRT, BUN/creatinine ratio > 20, urine protein/creatinine ratio > 3000 mg/g, Tacrolimus level > 12 ng/mL, CRP value > 5 mg/dL, arterial hypertension and diabetes mellitus.

To adjust for potential confounding factors, a multivariate linear regression model including independent variables TSH, age, gender, body mass index (BMI), THRT, BUN/creatinine ratio >20, urine protein/creatinine ratio >3000 mg/g, Tacrolimus level >12 ng/mL, CRP value >5 mg/dL, arterial hypertension and diabetes mellitus was established. Hereby, a significant association between ∆TSH and ∆eGFR was confirmed. Each unit (µIU/mL) increase in TSH led to a decrease in eGFR by 1.34 mg/mL [95% CI, −2.51 to −0.16; p = 0.03]. No association was found between single TSH values and eGFR at specific time points 12 and 24 months post transplantation and between single TSH values and course of eGFR over 12 months (Table 3). Statistical results remained virtually unchanged after exclusion of patients on thyroid hormone replacement therapy in the multivariate analysis (Supplementary Table S1).

No association was found between the thyroid hormones T3, FT4 and T4 and eGFR, neither at the individual time points 12 and 24 months post KTx nor when their differences between these time points (∆eGFR, ∆T3, ∆FT4, ∆T4) were analyzed (Table 4).

**Table 4:** Association between thyroid hormones and eGFR at various time points and periods post KTx.

Outcome	Exposure	Regression coefficient (95% CI) unadjusted	p-value	Regression coefficient (95% CI) adjusted*	p-value
eGFR	T3 at 12 mo	1.78 [−22.36 to 25.92]	0.88	−6.14 [−35.75 to 23.47]	0.68
	T3 at 24 mo	11.24 [−13.88 to 36.35]	0.37	2.61 [−34.62 to 39.83]	0.88
∆eGFR	T3 at 12 mo	0.14 [−13.92 to 14.20]	0.98	0.48 [−15.31 to 16.27]	0.95
	∆T3 from 12 to 24 mo	11.08 [−29.67 to 51.82]	0.57	−4.29 [−61.10 to 52.52]	0.86
eGFR	FT4 at 12 mo	14.14 [−13.60 to 41.88]	0.31	11.84 [−17.29 to 40.97]	0.41
	FT4 at 24 mo	−5.48 [−28.48 to 17.51]	0.63	−14.39 [−38.42 to 9.63]	0.23
∆eGFR	FT4 at 12 mo	−5.96 [−20.32 to 8.40]	0.41	−2.17 [−17.38 to 13.03]	0.78
	∆FT4 from 12 to 24 mo	−15.78 [−44.08 to 12.51]	0.25	−20.67 [−52.79 to 11.45]	0.16
eGFR	T4 at 12 mo	−0.07 [−0.42 to 0.29]	0.70	−0.16 [−0.62 to 0.29]	0.47
	T4 at 24 mo	−0.02 [−0.46 to 0.42]	0.92	0.11 [−0.40 to 0.62]	0.64
∆eGFR	T4 at 12 mo	−0.03 [−0.24 to 0.17]	0.74	0.11 [−0.12 to 0.35]	0.33
	∆T4 from 12 to 24 mo	−0.11 [−0.56 to 0.33]	0.60	−0.24 [−0.78 to 0.30]	0.32

∆eGFR = eGFR^24months^ − eGFR^12months^; ∆T3 = T3^24months^ − T3^12months^; ∆FT4 = FT4^24months^ − FT4^12months^; ∆T4 = T4^24months^ − T4^12months^. Numbers in brackets indicate the range.

Abbreviations: eGFR, estimated glomerular filtration rate; KTx, kidney transplantation; TSH, thyroid-stimulating hormone; mo, months. *Adjusted for age, gender, BMI, THRT, BUN/creatinine ratio > 20, urine protein/creatinine ratio > 3000 mg/g, Tacrolimus level > 12 ng/mL, CRP value > 5 mg/dL, arterial hypertension and diabetes mellitus.

Multivariate regression analysis for eGFR and TSH had a significant correlation with age, male gender, BMI and BUN/creatinine ratio 12 months after renal transplantation and with age and male gender 24 months post transplantation. Presence of diabetes was also associated with decreasing eGFR within 12 months (∆eGFR). Thyroid hormone replacement therapy, proteinuria with urine protein/creatinine ratio above 3000 mg/g, tacrolimus levels above 12 ng/mL, CRP values above 5 mg/mL and arterial hypertension as potential factors influencing thyroid hormones and eGFR had no significant effect at any time point and interval (Table 5).

**Table 5:** Multivariate linear regression analysis for eGFR and TSH at different time points and periods post KTx.

	eGFR at 12 months (95% CI)	p-value	eGFR at 24 months (95% CI)	p-value	∆eGFR/TSH at 12 mo (95% CI)	p-value	∆eGFR/∆TSH from 12 to 24 mo (95% CI)	p-value
TSH	0.01 [−2.11 to 2.12]	0.99	−0.19 [−2.08 to 1.71]	0.85	0.76 [−0.40 to 1.92]	0.20	−1.34 [−2.51 to −0.16]	0.03
Age	−0.38 [−0.51 to −0.26]	0.01	−0.38 [−0.51 to −0.25]	0.01	0.01 [−0.07 to 0.07]	0.96	0.01 [−0.06 to 0.07]	0.90
Male gender	6.71 [3.29 to 10.12]	0.01	6.28 [2.75 to 9.80]	0.01	−0.47 [−2.35 to 1.40]	0.62	−0.49 [−2.35 to 1.37]	0.60
BMI	−0.49 [−0.85 to −0.12]	0.01	−0.31 [−0.68 to 0.07]	0.11	0.18 [−0.02 to 0.38]	0.08	0.17 [−0.03 to 0.37]	0.09
THRT	−0.24 [−5.11 to 4.63]	0.92	0.41 [−4.65 to 5.47]	0.87	0.70 [−1.97 to 3.36]	0.61	0.26 [−2.43 to 2.94]	0.85
BUN/creatinine ratio > 20	5.40 [0.83 to 9.96]	0.02	3.27 [−1.44 to 7.97]	0.17	−2.30 [−4.81 to 0.20]	0.07	−2.22 [−4.70 to 0.26]	0.08
U: P/C > 3000 mg/g	7.69 [−8.11 to 23.49]	0.34	14.89 [−1.40 to 31.19]	0.07	6.87 [−1.78 to 15.53]	0.12	6.99 [−1.62 to 15.59]	0.11
Tacrolimus level > 12 ng/mL	2.48 [−7.21 to 12.16]	0.62	−1.08 [−11.07 to 8.91]	0.83	−3.17 [−8.47 to 2.14]	0.24	−3.52 [−8.78 to 1.75]	0.19
CRP value > 5 mg/dL	−3.71 [−14.24 to 6.82]	0.49	−6.36 [−17.24 to 4.52]	0.25	−2.61 [−8.38 to 3.16]	0.38	−2.12 [−7.88 to 3.65]	0.47
Arterial hypertension *	−0.63 [−1.70 to 0.43]	0.24	−0.47 [−1.57 to 0.63]	0.40	0.19 [−0.40 to 0.77]	0.53	0.17 [−0.41 to 0.75]	0.56
Diabetes mellitus	2.17 [−2.02 to 6.37]	0.31	−0.08 [−4.40 to 4.24]	0.97	−2.35 [−4.65 to −0.05]	0.05	−2.42 [−4.70 to −0.13]	0.04

∆eGFR = eGFR^24months^ − eGFR^12months^; ∆TSH = TSH^24months^ − TSH^12months^. Numbers in brackets indicate the range.

Abbreviations: BMI, body mass index; BUN, blood urea nitrogen; CRP, C-reactive protein; eGFR, estimated glomerular filtration rate; KTx, kidney transplantation; THRT, thyroid hormone replacement therapy; TSH, thyroid-stimulating hormone; U: P/C, urine protein/creatinine ratio, * quantified per number of antihypertensive substance class used to achieve current ESC/ESH blood pressure targets.

Furthermore, we analyzed whether the association between TSH and eGFR at different time points and time intervals post transplantation was modified by BUN/creatinine ratio. In samples stratified by BUN/creatinine ratio we observed some variability, but for none of the comparisons we found a significant interaction (Supplementary Table S2).

In the time period 12 to 24 months after transplantation 14 of 398 subjects developed pathological thyroid function parameters with TSH values between 3.77 and 7.11 µIU/mL, but normal T3, FT4 and T4 levels. In a regression analysis of these selected subjects, eGFR decreased by even 8.7% per unit (µIU/mL) TSH increase per year. However, the result was not significant due to the limited sample size.

## Discussion

This study was designed to analyze the association of changes of thyroid hormones and estimated glomerular filtration rate (eGFR) in CKD patients in the first two years after kidney transplantation. A significant association between the changes in TSH and eGFR levels in the course of the second year after transplantation between month 12 and 24 time points could be demonstrated.

Past studies in patients without kidney transplantation have already shown an association of overt and subclinical hypothyroidism (elevated TSH levels) with higher serum creatinine levels and lower eGFR, whereas hyperthyroidism was associated with lower serum creatinine and higher eGFR^4,7,10,14^. These changes were reversible with treatment: thyroid hormone replacement therapy in hypothyroidism led to improvement of renal function after regaining euthyroidism^13,19^. Some cohort studies showed an association of TSH levels, even within the normal range, with excretory kidney function. Higher TSH levels led to lower eGFR or higher prevalence of CKD^11,12,20^. The direct and indirect effects of thyroid hormones on renal function have already been described before. In addition to directly affecting renin-angiotensin system activity and ion channels and transporters of the kidney, which lead to upregulation of Na^+^-K^+^-2Cl^−^cotransporter and sodium uptake, the major impact on eGFR seems to be of rather indirect and functional nature. The effect of thyroid hormones throughout the whole body decreases systemic vascular resistance by enhanced nitric oxide synthase activity, increases cardiac output by enhanced inotropy and chronotropy and increases blood volume and hence, blood pressure. Altogether these effects lead to an increased renal plasma flow, reduced renal vascular resistance and as a result to higher GFR^5,6,21–23^. Unlike in overt hypothyroidism, treatment of subclinical hypothyroidism remains debatable. It is generally recommended by the European and American Thyroid Associations to start treatment with levothyroxine (T4) at TSH levels above 10 µIU/mL and in patients under 65 years of age at TSH levels between 7 and 10 µIU/mL due to higher cardiovascular mortality^24–26^. Hormone replacement below these levels is symptom-dependent and controversial^27^. Beyond that, there are no statements about treatment of euthyroid patients. Patients with a kidney transplant are considered CKD patients, even if their GFR is within the normal range and recommendations do not distinguish between CKD patients with or without kidney transplantation.

With regard to treatment of subclinical hypothyroidism, two studies by Shin *et al*. are of note.

One retrospective study with 309 patients showed that thyroid hormone therapy preserved renal function better and was also an independent predictor of renal outcome in patients with CKD. In a subsequent study in 113 CKD patients with subclinical hypothyroidism, the decline of eGFR was attenuated by hormone replacement treatment^15,16^. Only a few studies investigated the interactions between thyroid hormones and transplanted kidneys. A study by Junik *et al*. proved that renal transplantation might have an impact on thyroid hormones, especially on T3 concentrations^17^. And while low T3 might be an independent risk factor for renal graft failure^28,29^, T3 treatment did not improve graft survival or function^30^.

Since TSH levels seem to be associated with eGFR and CKD^10^, euthyroid patients might benefit from low TSH levels^11,12^. Yet, there have been no studies examining the association of TSH levels in euthyroid CKD patients with kidney graft function. In our study, we included 398 euthyroid patients who received a kidney transplant between 2003 and 2016. Our data show that in the second post-transplant year an increase of TSH levels over 12 months (∆TSH) results in an annual decrease of eGFR of 1.34 mL/min per unit (µIU/mL) TSH (∆eGFR). However, mean values of eGFR and TSH do not show any differences at single time points 1 year and 2 years post transplantation. Thus, the individual change of TSH values over time and its effect on eGFR is of particular significance. Our data indicate that even within the euthyroid range, decreasing thyroid function, as indicated by an increase in TSH within the reference range, is associated with decreasing transplant function, independently of age, gender, body mass index and thyroid hormone replacement therapy. We also included proteinuria as well as elevated tacrolimus levels, CRP values, arterial hypertension and diabetes mellitus as factors that may influence thyroid hormones and eGFR in our analysis. Of note, a high BUN/creatinine ratio in serum as sign of hypovolemia was associated with reduction of eGFR, but not with TSH values, which was tested in an interaction analysis. In the present study patients with subclinical or overt hypothyroidism were excluded, but data were not censored for the development of subclinical hypo- or hyperthyroidism within the observation period. In a subanalysis, only 14 subjects developed subclinical hypothyroidism within 12 months. These subjects with particularly high increase in TSH showed a clearly higher decrease in eGFR of 8.7% per unit (µIU/mL) increase of TSH, which supports our main study results, although eGFR decrease in the euthyroid study population was lower. Although this finding is not significant due the limited number of patients affected, it supports our basic hypothesis. Forty-eight subjects who were euthyroid due to THRT were excluded in a subanalysis and statistical results remained virtually unchanged after exclusion of these patients in the multivariate analysis. Our study results showed no association between T3, FT4 and T4 and eGFR, neither at individual time points 12 and 24 months post KTx nor over time.

The main limitation of this study is the retrospective design. Furthermore, follow-up was limited to 2 years after transplantation. However, our study was strengthened by a large sample size of 398 subjects and a well-characterized cohort of euthyroid CKD patients in the early post-transplantation phase, which led to clear results: the cross-sectional analysis of individual thyroid and renal function parameters at selected times 12 and 24 months after KTx showed no association. However, the assessment of the changes in TSH and eGFR during the second year after transplantation showed a significant dependence between thyroid and transplanted kidney functions: as TSH levels increase over time, eGFR tends to decrease. These results are consistent with previous data demonstrating a decrease in eGFR by 1.9% for each 1 µIU/mL increase in TSH^11^. To further verify a causal association between thyroid function parameters and eGFR in kidney transplant CKD patients, prospective randomized placebo-controlled trials are underway to investigate whether thyroid hormone replacement therapy targeting low-normal TSH levels can enhance eGFR.

## Supplementary information

Supplementary Information.

## Data Availability

Data sets analyzed in the present study can be obtained on request from the corresponding author.
