# Supplementary material for: Effect of Thyroid Hormones on Kidney Function in Patients after Kidney Transplantation
Source: Sci Rep. 2020 Feb 7;10:2156. doi: 10.1038/s41598-020-59178-x (PMC7005730; doi:10.1038/s41598-020-59178-x)
Supplement: Supplementary file 1 — Supplementary Information. [file 41598_2020_59178_MOESM1_ESM.docx]

# Effect of Thyroid Hormones on Kidney Function in Patients after Kidney Transplantation

# Benjamin Schairer^1^, Viktoria Jungreithmayr^2^, Mario Schuster^2^, Thomas Reiter^1^, Harald Herkner^3^, Alois Gessl^4^, Gürkan Sengölge^1*^, Wolfgang Winnicki^1^

^1^ Department of Internal Medicine III; Division of Nephrology and Dialysis, Medical University of Vienna, Austria

^2^ Department of Pharmacology and Toxicology, University of Vienna, Austria

^3^ Department of Emergency Medicine, Medical University of Vienna, Austria

^4^ Department of Internal Medicine III, Division of Endocrinology, Medical University of Vienna, Austria

^*^ Corresponding author

E-mail address: guerkan.sengoelge@meduniwien.ac.at

Supplementary Table S1. Association between TSH and eGFR at various time points and periods post KTx without 48 patients on thyroid hormone replacement therapy (n=350)

| **Outcome** | **Exposure** | **Regression coefficient (95% CI)**  **unadjusted** | **p-value** | **Regression coefficient (95% CI)**  **adjusted*** | **p-value** |
| --- | --- | --- | --- | --- | --- |
| eGFR | TSH  at 12 mo | -0.18 [-2.63 to 2.26] | 0.88 | -0.66 [-2.98 to 1.67] | 0.58 |
|  | TSH  at 24 mo | -0.13 [-2.20 to 1.94] | 0.90 | -0.20 [-2.18 to 1.78] | 0.84 |
| ∆eGFR | TSH  at 12 mo | 1.31 [0.07 to 2.54] | 0.04 | 1.18 [-0.09 to 2.45] | 0.07 |
|  | ∆TSH  from 12 to 24 mo | -2.09 [-3.39 to -0.79] | 0.01 | -2.11 [-3.43 to -0.79] | 0.01 |

∆eGFR = eGFR^24months^ – eGFR^12months^; ∆TSH = TSH^24months^ – TSH^12months^. Numbers in brackets indicate the range.

Abbreviations: eGFR, estimated glomerular filtration rate; KTx, kidney transplantation; TSH, thyroid-stimulating hormone; mo, months. *Adjusted for age, gender, BMI, BUN/creatinine ratio >20, urine protein/creatinine ratio >3000mg/g, Tacrolimus level > 12ng/mL, CRP value > 5mg/dL, arterial hypertension and diabetes mellitus.

**Supplementary Table S2. Interaction of BUN/creatinine ratio with the effect of TSH on eGFR at various time points**

|  | **TSH at 12 months on eGFR (95% CI)** | **p-value for interaction** | **TSH at 24 months on eGFR (95% CI)** | **p-value for interaction** | **TSH at 12 months on ∆eGFR (95% CI)** | **p-value for interaction** | **∆TSH on ∆eGFR (95% CI)** | **p-value for interaction** |
| --- | --- | --- | --- | --- | --- | --- | --- | --- |
| If BUN/creatinine ratio <20 | -0.51 [-2.8 to 1.8] | 0.46 | -0.58 [-2.6 to 1.4] | 0.21 | 1.22 [0.1 to 2.4] | 0.19 | -1.3 [-2.3 to -0.3] | 0.56 |
| If BUN/creatinine ratio >20 | 1.33 [-4.5 to 7.1] |  | 0.71 [-5.3 to 6.8] |  | -0.48 [-5.3 to 4.4] |  | -3.40 [-10.1 to -3.3] |  |

∆eGFR = eGFR^24months^ – eGFR^12months^; ∆TSH = TSH^24months^ – TSH^12months^. Regression coefficient and 95% confidence intervals are given. Numbers in brackets indicate the range.

Abbreviations: BUN, blood urea nitrogen; CI, confidence interval; eGFR, estimated glomerular filtration rate; TSH, thyroid-stimulating hormone.
